# Jejunal diverticulitis secondary to a gastrointestinal stromal tumor: A case report

**DOI:** 10.1016/j.ijscr.2021.106291

**Published:** 2021-08-08

**Authors:** Douglas Chung

**Affiliations:** Campbelltown Hospital, Therry Road, Campbelltown, NSW 2560, Australia

**Keywords:** Case report, Gastrointestinal stromal neoplasm, Jejunal disease, Diverticulitis, Intestinal perforation

## Abstract

**Introduction:**

The common manifestations of gastrointestinal stromal tumors (GIST) are well established. However, jejunal diverticulosis is an uncommon phenomenon to be associated with this lesion, with its rarity compounded by the relative difficulty associated with its diagnosis. Limited literature is available on this topic. This article examines one such case of jejunal diverticulitis as a result of a GIST, and the intervention of said disease.

**Case presentation:**

A 69 year old lady presented with abdominal pain, vomiting, and low grade fevers, on a background of ulcerative colitis. She was peritonitic, raising concerns of an acute abdomen. Her imaging identified an intra-abdominal contained perforation, prompting a transfer to theatres overnight for a laparotomy, which identified a jejunal diverticulum, which resembled a contained perforation. This was resected, and sent for histopathological analysis, identifying the lesion as a GIST.

**Discussion:**

Unlike other forms of jejunal diverticular disease, those arising from GISTs tend to present perforated, necessitating resection. This disease displays a tendency towards formation on the anti-mesenteric border of the small bowel. Additionally, this particular form of GIST shows macroscopic and histopathological uniformity across reported cases to date despite significant geographical disparity.

**Conclusion:**

A scant number of case reports worldwide have identified jejunal diverticulitis from GISTs. We suggest diverticula be excised if perforation is suspected, while incidental findings of such be left untouched. However, overall management should be undertaken at the discretion of the operating surgeon.

## Highlights

• GIST forming a jejunal diverticulum is an uncommon manifestation.
• Usually present perforated unlike other forms of diverticula, which are indolent.
• Jejunal diverticula should be resected when perforation suspected.
• Histopathological and macroscopic homogeneity across the globe.

## 1 Introduction

The common manifestations of gastrointestinal stromal tumours (GIST) are well established, typically presenting as exophytic lesions in the stomach, and less commonly in the small intestine [1], [2]. Jejunal diverticulosis is a significantly less common phenomenon, with quoted incidence rates ranging from 0.02% on standard imaging modalities up to 4.6% on autopsy studies [3]. Its formation from a gastrointestinal stromal tumor (GIST) is rare, with scant literature available limited to a handful of case reports [2], [4]. Its rarity is compounded by the difficulty associated with establishing its diagnosis. This article examines one such case of jejunal diverticulitis as a result of a GIST, its management in the hospital setting, and the interventions undertaken.

## 2 Methods

A retrospective case review was undertaken of in keeping with the SCARE 2020 criteria [5].

### 2.1 Case presentation

A 69 year old Lebanese Australian lady was brought in by ambulance to hospital with generalised abdominal pain of 24 h. This was associated with nausea, vomiting, constipation of 3 days and low grade fevers of 38.0. Her medical history was positive for ulcerative colitis, for which she was on azathioprine, mesalazine, and a biological agent; chronic lymphocytic leukaemia which was being monitored; and hypertension. There was no history of smoking or alcoholism. She was retired and lived in a large household with multiple family members.

**Fig. 1:**
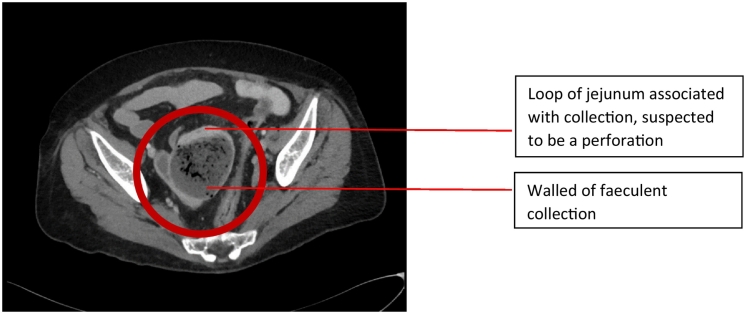
Axial view of CT scan at presentation.

**Fig. 2:**
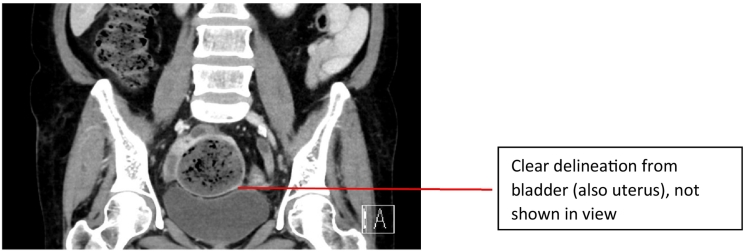
Coronal view of CT scan at presentation.

Her abdominal examination showed signs of peritonitis. Despite this, she remained haemodynamically stable, though borderline hypotensive with IV fluid resuscitation. Her inflammatory markers were chronically raised, making interpretation difficult. This prompted an urgent CT scan, which revealed a faeculent walled off collection measuring 56x58x60mm abutting the small bowel loops in her pelvis (Fig. 1, Fig. 2). The decision was made to undertake an urgent laparotomy, due to suspicion of a perforated viscus. After adequate resuscitation and pre-operative workup, the patient was urgently transferred to the operating theatres. She was commenced on intravenous ampicillin, gentamicin, and metronidazole (the standard antibiotics of choice for intra-abdominal sources of infection).

The consultant surgeon on call undertook the procedure, assisted by the evening general surgical registrar. A laparotomy was performed, which revealed what appeared to be a contained perforation attached to the proximal jejunum on its anti-mesenteric border. Its contents were frankly faeculent, and a decision was made to resect a 12 cm section of small bowel including the perforation. Primary hand-sewn anastamosis of the remaining bowel was preferred over stapled anastamosis due to the thickness of the involved bowel wall and adjacent mesentery. Other notable intraoperative features include a number of small simple jejunal diverticula adjacent to the site of primary pathology; a small amount turbid fluid within the abdomen; and scant fibrinous exudate scattered amongst the remainder of her small bowel. Given the absence of complications, a decision was made to leave the incidental adjacent diverticula in situ.

As the patient was haemodynamically stable during surgery, she was transferred to the surgical ward post-operatively. The nasogastric tube was left in place until resolution of post-operative ileus, then removed and enteral feeding resumed. IV antibiotics were continued for the first 3 days post-operative days only. The remainder of her inpatient admission was unremarkable, and she was discharged home 1 week post-operatively, to follow up with her surgeon and gastroenterologist in 6 weeks for continued care.

Histopathological examination of the resected specimen showed patchy areas epithelioid cells with moderate nuclear atypia, alongside occasional multinucleated giant cells within the diverticulum. The wall thickness was between 1 mm and 10 mm. The neck comprised of solid areas of bland spindle cells with elongated nuclei. The diverticulum itself arose from the muscular layer of the wall, coated internally with a complete internal mucosa. While it pushed the overlying serosa outwards, there was no evidence of serosal perforation associated with this lesion. Immunohistochemistry revealed the lesion to be KIT (CD117), CD34, and DOG1positive. The lesion was identified as a GIST forming a true jejunal diverticulum, of a mixed spindle cell type with a minor epithelioid component.

## 3 Discussion

This particular case marks a divergence from the usual indolent course of jejunal diverticulosis [3], [6], likely owing to its underlying lesion. Various difficulties in establishing the underlying pathology make the diagnosis of this disease complicated [3], [6], [7]. While most cases of diverticulosis are asymptomatic, patients may present with an array of symptoms, including recurrent diverticulitis, intussusception, bleeding [6], [7], and obstruction [8]. Acute presentations to the emergency department may be caused by massive haemorrhage [6] and perforation [9].

**Fig. 3:**
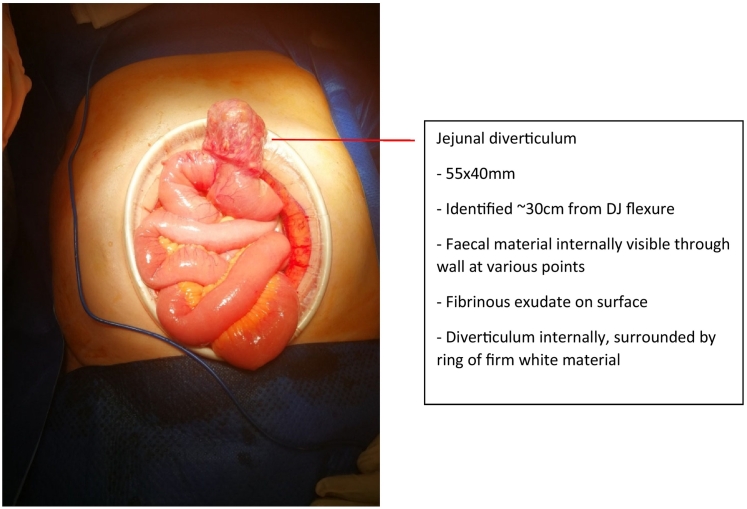
Intraoperative photo of lesion.

The patient's clinical presentation was likely secondary to diverticulitis. Based on the histopathological findings, the disease process discussed in this case represents a true jejunal diverticulum as opposed to a perforation, despite the appearance of the lesion in Fig. 3 and imaging findings to the contrary. This illustrates the difficulty of assessing the presence of complications in this disease.

Few case reports [2], [4] of perforations of such diverticula have been published. These reports suggest resection is required in the event of perforation. However, the presence of multiple other concurrent jejunal diverticula in our patient suggest the possibility of indolent disease, which could not be identified on imaging. Indeed, it has been suggested that the disease is hard to identify even on post-mortem examinations [3]. The imaging modalities available in the diagnosis of jejunal diverticulosis are barium small bowel series [10], CT scans [10], and enteroclysis [11]. These modalities are either poorly sensitive or complex and time consuming. This suggests a high likelihood of the disease being underappreciated.

In the acute presentations described above emergent surgery is generally indicated [6]. The approach to this patient's management was in keeping with this recommendation, and with local standard practice of a suspected perforated viscus. Another consideration is the size of the collection, as local guidelines have advised non-operative management is unlikely to succeed in lesions of greater than 5 cm along any axis. These guidelines are extrapolated from available evidence [12] suggesting the likelihood of antibiotic therapy failure increases from 3 cm in diameter.

Another interesting point of note is the macroscopic and microscopic descriptions of the lesion, which bear a striking resemblance to those described in other case reports [2], [4]. Significant uniformity is observed despite the wide range of nationalities and ethnicities of the patients, in this case Japanese, Belgian, and Lebanese. All identified cases exhibit identical characteristics in being found on the anti-mesenteric border, which differ from typical jejunal diverticulosis which is found along the mesenteric border [3], usually within the mesentery itself as weaknesses in the wall from entry the vasa recta.

Lack of data limits our recommendations on management to extrapolation of pre-existing cases. Resection of jejunal diverticula is recommended in the event of suspected perforation. A method of identifying the presence of perforation would have been ideal in guiding intraoperative decision making. However, this case did not identify any findings that would have suggested the uncomplicated nature of the disease prior to hisopathological examination. A larger sample size and further research is likely to be required in this area. Incidental findings of mesenteric diverticula should not be operated on given their usual course, particularly in the acute setting.

### 3.1 Patient perspective

The patient has recovered uneventfully following this event. No significant issues were encountered in the immediate follow up period following the event, and she has not had any recurrence of her symptoms. She was understanding of our decision making process at the time of her presentation.

## 4 Conclusion

This disease shows remarkable macroscopic uniformity across the reported cases to date despite their geographical disparity. A scant number of case reports worldwide have identified this jejunal diverticulitis from GISTs, insufficient to provide a strong evidence based guideline. This case report identifies the difficulty of intraoperative assessment of complications associated with this lesion. We suggest suspected perforated diverticula be excised, while incidental findings of such be left untouched. However, overall management should be undertaken at the discretion of the operating surgeon.

## Sources of funding

This research did not receive any specific grant from funding agencies in the public, commercial, or not-for-profit sectors.

## Ethical approval

The Southwestern Sydney Local Health district was contacted with regards to this case report, and advised that case reports are handled by local hospital policy. The hospital Director of Medical Services and Head of the Surgical Department have separately verified that a consent form from the patient provided by the local health district was sufficient. The aforementioned form has been completed.

## Consent

Written informed consent was obtained from the patient for publication of this case report and accompanying images. A copy of the written consent is available for review by the Editor-in-Chief of this journal on request.

## Research registration

N/A.

## Guarantor

The corresponding author is the guarantor of this manuscript.

## Provenance and peer review

Not commissioned, externally peer-reviewed.

## Credit authorship contribution statement

Case report design, data collection, data interpretation, writing the paper.

## Declaration of competing interest

No conflicts of interest were identified in the writing of this case report.
